# Efficacy of combination therapy with GABA, a DPP-4i and a PPI as an adjunct to insulin therapy in patients with type 1 diabetes

**DOI:** 10.3389/fendo.2023.1171886

**Published:** 2023-05-24

**Authors:** Alexander Rabinovitch, Daniil Koshelev, Francisco Alejandro Lagunas-Rangel, Liudmila Kosheleva, Tali Gavra, Helgi B. Schiöth, Shmuel Levit

**Affiliations:** ^1^ Kelowna, BC, Canada; ^2^ Department of Surgical Sciences, Functional Pharmacology and Neuroscience, Uppsala University, Uppsala, Sweden; ^3^ Levicure LTD, Rishon Lezion, Israel; ^4^ Research Unit, Assuta Medical Centers, Tel Aviv, Israel; ^5^ Diabetes and Metabolism Institute, Assuta Medical Centers, Tel Aviv, Israel

**Keywords:** type 1 diabetes, insulin, GABA, DPP-4i, PPI

## Abstract

**Introduction:**

The purpose of this retrospective clinic chart review study was to determine the potential of a combination therapy (CT) consisting of γ-aminobutyric acid (GABA), a dipeptidyl peptidase-4 inhibitor (DPP-4i), and a proton pump inhibitor (PPI) to improve glycemic control as an adjunct to insulin therapy in patients with type 1 diabetes (T1D).

**Research design and methods:**

Nineteen patients with T1D on insulin therapy were treated with additional CT in oral form. Fasting blood glucose (FBG), HbA1c, insulin dose-adjusted HbA1c (IDA-A1c), daily insulin dose, insulin/weight ratio (IWR), and fasting plasma C-peptide were measured after 26-42 weeks of treatments.

**Results:**

FBG, HbA1c, IDA-A1c, insulin dose and IWR were all significantly decreased while plasma C-peptide was significantly increased by the CT. Treatment outcomes were further analyzed by separation of the 19 patients into two groups. One group started on the CT within 12 months of insulin treatment (early therapy, 10 patients) and another group started on this therapy only after 12 months of insulin treatment (late therapy, 9 patients). FBG, IDA-A1c, insulin dose, and IWR decreased significantly in both the early and late CT groups, however to a better extent in the early therapy group. Moreover, plasma C-peptide increased significantly only in the early therapy group, and 7 of the 10 patients in this group were able to discontinue insulin treatment while maintaining good glycemic control to study end compared with none of the 9 patients in the late therapy group.

**Conclusion:**

These results support the concept that the combination of GABA, a DPP-4i and a PPI as an adjunct to insulin therapy improves glycemic control in patients with T1D, and that the insulin dose required for glycemic control can be reduced or even eliminated in some patients receiving this novel therapy.

## 1 Introduction

Type 1 diabetes (T1D) is a disease characterized by a lack of insulin production by pancreatic islet β-cells due to their autoimmune destruction (1–3). A recent modeling study demonstrates that the burden of T1D in 2021 is considerable and is predicted to rise rapidly, especially in resource-limited regions with the highest incidence and prevalence occurring in adults (4). T1D develops mainly in childhood or adolescence, and, to date, there is no known way to reverse it (5). Treatment options are very limited, and most patients rely solely on insulin therapy (6). In addition, the high costs of new technologies based on advanced insulin treatments reduce their universal accessibility (7). Procedures such as islet or pancreas transplantation cannot be considered consistently reliable and scalable treatment options, due to a number of limiting factors including their extreme complexity, donor availability, requirement for lifelong immunosuppression, and high cost (8).

Considerable attention has been devoted to discovering a treatment that can provide physiological glycemic control and minimize or remove the need for exogenous insulin. Numerous studies attempting to prevent or reverse T1D have centered on immune suppression or regulation (9). The most promising treatment options have focused on the anti-CD3 antibody Teplizumab, which has been reported to provide a 3-year delay in the clinical diagnosis of T1D (10, 11), but arrest of continued β-cell loss remains unattained.

Another avenue of research in T1D is the repurposing of already-approved medications, particularly those that have shown promise in preserving β-cell function in pre-clinical studies, including gastrin, dipeptidyl peptidase-4 inhibitors (DPP-4i), glucagon-like peptide 1 receptor agonists (GLP-1RA), γ-aminobutyric acid (GABA), glutamic acid decarboxylase 65 antigen (GAD-alum) and proton pump inhibitors (PPI). Preclinical investigations using human islet cells and animal T1D models have reported on the effectiveness of DPP-4i to protect β-cells from immune destruction (12, 13). In addition, a systematic evaluation of the use of DPP-4i in five randomized, controlled clinical trials revealed lower HbA1c levels and confirmed the safety and tolerability of DPP-4i in T1D (14). Recently, it was reported that the combination of GABA and GAD-alum, in a 12-month, double-blind, randomized T1D pediatric trial, significantly reduced fasting and meal-stimulated serum glucagon, but preservation of the C-peptide response was not attained, however the safety of GABA was confirmed (15). We have recently reported that a three-drug combination therapy consisting of the neurotransmitter GABA, a DPP-4i and a PPI can prevent and reverse diabetes in non-obese diabetic (NOD) mice, an animal model for human T1D, whereas all two-drug combinations were less or not effective (16). The objective of the study reported here was to determine whether glycemic control in patients with T1D could be improved by adding a combination of GABA, DPP-4i, and PPI to a standard insulin treatment regimen in these patients, in a real-world clinical setting.

## 2 Methods

### 2.1 Study design and subjects

A retrospective clinic chart review study was performed to evaluate the impact of a combination therapy with GABA, a DPP-4i (sitagliptin or saxagliptin) and a PPI (omeprazole) on fasting blood glucose control, HbA1c, insulin requirements, and fasting plasma C-peptide levels in people with T1D at the Diabetes and Metabolism Institute, Assuta Medical Centers (Tel Aviv, Israel). The study was approved by the Helsinki Ethics Committee of Assuta Medical Centers (Tel Aviv, Israel) with a protocol number ASMC-0076-22.

All electronic medical records (EMR) from 2015-2022 were examined to identify T1D patients treated with insulin and receiving adjunct therapy with all three drugs (GABA, DPP-4i, and PPI) concurrently and for a duration of 26 weeks or more. Individual patient EMRs were examined in order to confirm the diagnosis of T1D, based upon the diagnostic criteria of the American Diabetes Association (ADA) and the European Association for the Study of Diabetes (EASD) (17). Confirmation of the T1D diagnosis included the detection of glutamic acid decarboxylase 65 (GAD) autoantibodies and/or a fasting plasma C-peptide level <200 pmol/L (17). A total of nineteen patients met these criteria for inclusion in the retrospective analysis of the data here reported.

The starting date of the addition of the combination therapy (CT) consisting of GABA, DPPI-4i and PPI to insulin therapy was established using prescription records. Continuous use of CT, concurrent with insulin use, during the 26-42 weeks of study was confirmed by review of the clinical records.

### 2.2 Data analysis

Data analysis involved comparing laboratory values for each patient at baseline prior to CT and corresponding values for that patient at their follow-up visit after ≥26 weeks of CT administration. Depending on the patient’s availability and the collection of all relevant laboratory samples, the follow-up visits took place between 26 and 42 weeks of continuous CT administration. Changes over time (baseline to clinic visit at >26 weeks) in the study population of nineteen patients were collected and analyzed for the following parameters: weight (kg), BMI (index score), FBG (mg/dL), HbA1c (%), IDA-A1c (%), insulin dose (Units/day), insulin/weight ratio (IWR, Units/kg/day), and fasting plasma C-peptide (pmol/L).

### 2.3 Statistical methods

For the statistical evaluation of the data obtained, specialized software GraphPad Prism v 9.4 (GraphPad Software, San Diego, California, USA) was used. The normality of the distribution of parameter values in the study groups was assessed using the Shapiro-Fork criterion. Differences between the before and after treatment values were assessed by using a paired comparison using Student’s t-test (if the distribution of values were close to normal) or by Wilcoxon’s W-test (if the distribution of values differed from normal).

## 3 Results

### 3.1 Patient characteristics

The nineteen patients receiving insulin and concurrent adjunct combination therapy (CT) consisting of GABA, a DPP-4i and a PPI were confirmed to have T1D. The diagnosis of T1D was based upon all available data in the clinic chart records, including, glucose >20 mmol/L, ketoacidosis, other clinical features (such as polydipsia and polyuria) at the onset, and records of insulin dependence on admission to clinic. Additionally, T1D diagnosis was confirmed based upon ADA and EASD guidelines regarding islet autoantibodies, followed by fasting plasma C-peptide levels if islet autoantibody tests were negative or absent (17). Accordingly, GAD autoantibodies were present in 14 patients and fasting plasma C-peptide levels were <200 pmol/L in another 5 patients in whom GAD autoantibodies were negative (3 patients) or data were absent (2 patients). Fasting plasma C-peptide levels were measured in all 19 patients at the study start (baseline), but unfortunately were not measured again in 2 patients at study end, so that 2 patients out of the total of 19 did not have a recorded pair of fasting C-peptide measurements. **Table 1** shows that Diabetes was diagnosed and treated with insulin at a mean age of 32 years (range 9-57 years). However, the CT was added to insulin treatment only after a wide time interval following the start of insulin treatment (mean 81 months, range 0.3-420 months). Therefore, at the start of the addition of CT to insulin therapy, patients had a mean age of 38 years (range 17-83 years).

**Table 1:** Characteristics of patients enrolled in the study.

Characteristic	Total patients (n = 19)	Early therapy (n = 10)	Late therapy (n = 9)
Male/Female	12/7	7/3	5/4
Age of patients at diabetes onset and the start of insulin treatment (years)	32 ± 13 (9-57)	31 ± 13 (17-57)	33 ± 14 (9-56)
Age of patients at the start of combination drug therapy (years)	38 ± 18 (17-83)	31 ± 13 (17-58)	47 ± 19 (27-83)
Duration of insulin treatment before the start of the combination drug therapy (months)	81 ± 124 (0.3-420)	3.1 ± 3.5 (0.3-11)	168 ± 135 (41-420)
Duration of combination drug therapy (weeks)	31 ± 6 (26-42)	32 ± 6 (27-42)	29 ± 5 (26-41)

Patients were treated with a three-drug combination of GABA, a DPP-4i and a PPI added to insulin therapy. Values are shown for the total patient population (n =19) and for two subgroups of patients: i) those that started the combination drug therapy within 12 months after the start of insulin treatment (Early therapy, n = 10 patients) and ii) those that started the combination drug therapy more than 12 months after the start of insulin treatment (Late therapy, n = 9 patients). Values are means +/- SD and ranges are shown in parentheses.

### 3.2 GABA, DPP-4i and PPI therapy characteristics

The components of the CT were prescribed in the following dose ranges: GABA (GABA Solgar) 1000-2000 mg/day (500 mg capsules taken 2-4 times a day); a DPP-4i, sitagliptin (Januvia) 50-100 mg/day in 16 patients or saxagliptin (Onglyza) 5 mg/day in 3 patients; and a PPI omeprazole (Omeprazole Teva) 20-40 mg/day. All medications were administered orally. The prescribed doses of sitagliptin, saxagliptin, and omeprazole did not exceed the standard FDA recommended amounts.

### 3.3 Study outcomes in the total patient population

FBG, HbA1c, IDA-A1c, daily insulin dose, and IWR were all significantly decreased after treatment for 26-42 weeks with GABA, a DPP-4i and a PPI added to insulin therapy compared with baseline levels before therapy, and fasting plasma C-peptide was significantly increased (**Table 2**).

**Table 2:** Effects of combination drug therapy with GABA, a DPP-4i and a PPI in the total patient population (n=19).

Characteristic	Before therapy	After therapy	n (pairs)	p
Weight (Kg)	73.7 ± 13.9	72.5 ± 13.2	19	0.113
BMI (index score)	24.5 [21.1; 25.7]	23.9 [20.9; 25.7]	19	0.057
FBG (mg/dL)	162 ± 46	126 ± 31	19	0.002
HbA1c (%)	8.5 [6.5; 11.9]	6.4 [5.9; 7.5]	19	0.003
IDA-A1c (%)	10.4 ± 2.9	7.4 ± 1.6	19	0.001
Insulin dose (Units/day)	25.3 ± 19.5	13.3 ± 13.0	19	0.001
IWR(Units/Kg/day)	0.35 ± 0.25	0.19 ± 0.19	19	0.001
C-peptide (pmol/L)	139 [33; 200]	195 [33; 470]	17	0.027

Data are means ± SD if the distribution of values was close to normal, or medians with interquartile width in parenthesis if the distribution of values differed from normal. P-values are levels of significant paired differences between values before and after the triple drug combination therapy, for the number of patients (n) indicated.

### 3.4 Characteristics of patients divided into two subgroups

Study outcomes were examined further by separating the total patient population (19 patients) into two subgroups: i) patients administered CT within 12 months after the start of insulin treatment (early therapy, 10 patients) and ii) patients administered CT more than 12 months after the start of insulin treatment (late therapy, 9 patients). **Table 1** shows that the ages of diabetes onset and accompanying insulin treatment were similar in the early and late CT subgroups (means, 31 and 33 years). However, the duration of insulin treatment before the administration of CT in the early therapy group (mean 3.1 months, range 0.3-11 months) was much shorter than that in the late therapy group (mean 168 months, range 41-420 months).

### 3.5 Study outcomes in the two subgroups of patients

**Figure 1:**
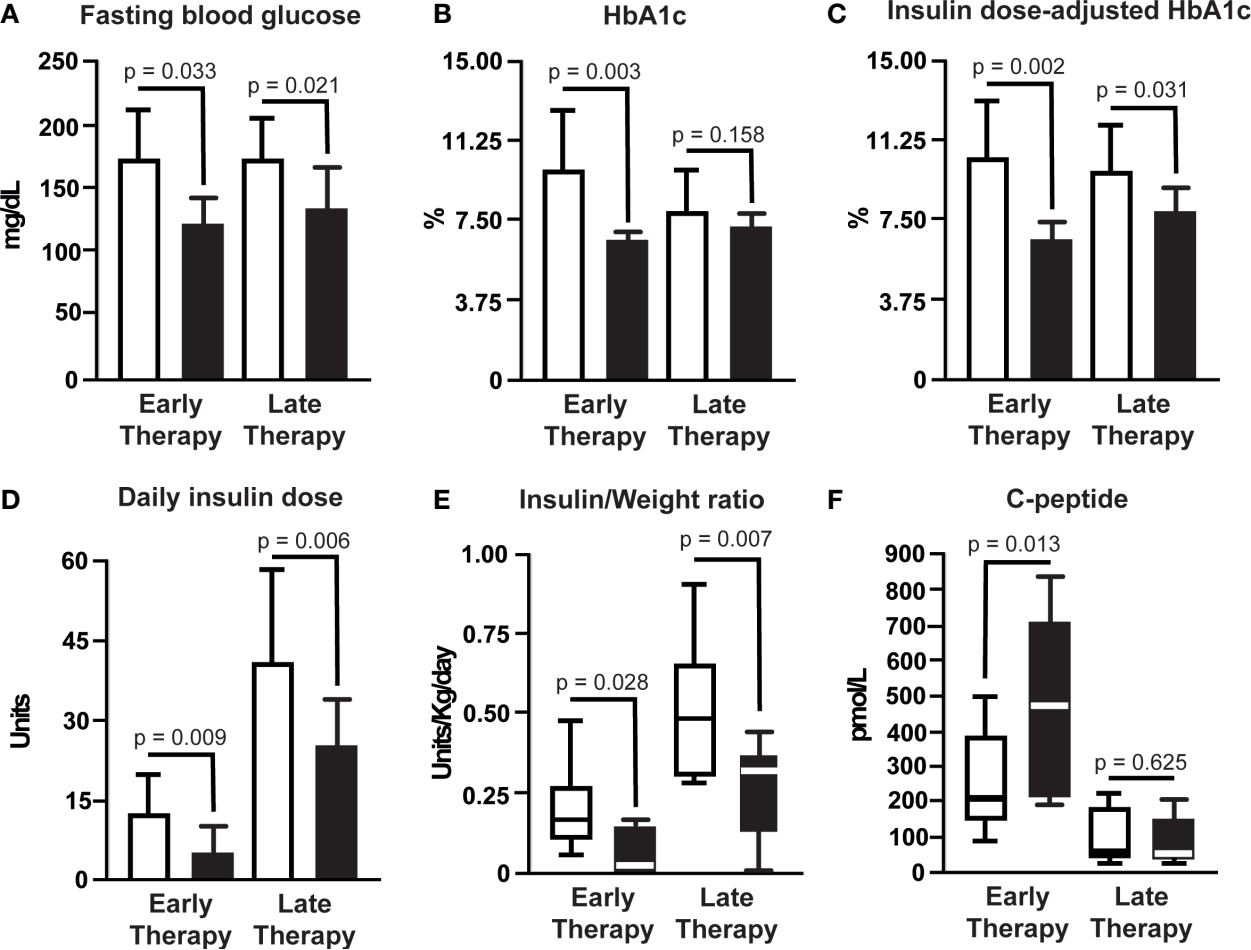
Fasting blood glucose **(A)**, HbA1c **(B)**, insulin dose-adjusted HbA1c **(C)**, daily insulin dose **(D)**, insulin/weight ratio **(E)** and fasting plasma C-peptide **(F)** values are shown before (white bars) and after (black bars) treatment with the combination of GABA, a DPP-4i, and a PPI within 12 months of insulin treatment in 10 patients (early therapy) and more than 12 months after the start of insulin treatment in 9 patients (late therapy). Data are means ± SD if the distribution of values was close to normal, or medians with interquartile width if the distribution of values differed from normal. P-values are levels of significant paired differences between values before and after therapy for the numbers of patients indicated above, except for C-peptide values in 8 of the 10 patients in the early therapy group.

Study outcomes were determined at similar times after starting CT in the early therapy group (mean 32 weeks, range 27-42 weeks) and in the late therapy group (mean 29 weeks, range 26-41 weeks) (**Table 1**). Study outcomes in the two groups of patients are shown in **Table 3** and illustrated in **Figure 1**. In the early therapy group, body weight and BMI were slightly but significantly decreased, and in the late therapy group, body weight and BMI were similar before and after therapy. FBG levels were significantly decreased in both the early (p=0.033) and late therapy (p=0.021) groups. HbA1c was significantly decreased in the early therapy group (p=0.003), but not significantly in the late therapy group (p=0.158). However, IDA-A1c levels were significantly decreased in both the early therapy group (p=0.002) and the late therapy group (p=0.031). At baseline before therapy, the daily insulin dose in the early therapy group (mean, 12.5 units) was significantly lower than in the late therapy group (mean, 39.6 units, p=0.005) and the IWR in the early therapy group (median,0.15 units/kg/day) was significantly lower than in the late therapy group (median, 0.49 units/kg/day, p=0.007). Daily insulin doses and IWR decreased significantly after CT in both groups. At baseline before therapy, the fasting plasma C-peptide level in the early therapy group (median, 190 pmol/L) was significantly higher than in the late therapy group (median, 33 pmol/L, p=0.004). Furthermore, the C-peptide level was significantly increased after CT in the early therapy group, from a median of 190 to 470 pmol/L (p=0.013), whereas the C-peptide level remained very low in the late therapy group (median, 33 pmol/L, p=0.625).

**Table 3:** Effects of combination drug therapy with GABA, a DPP-4i and a PPI in two subgroups of patients, those with early and those with late therapy.

Characteristic	Early therapy				Late therapy			
	Before therapy	After therapy	n (pairs)	p	Before therapy	After therapy	n (pairs)	p
Weight (Kg)	74.2 [58.3; 84.3]	71.9 [56.8; 82.9]	10	0.039	75 [68.8; 82.5]	76 [65.8, 83.3]	9	0.718
BMI (index score)	23.5 [19.3; 26.2]	22.9 [18.9; 24.6]	10	0.016	24.6 [22.5; 25.9]	23.9 [22.6; 25.9]	9	1.000
FBG (mg/dL)	162 ± 55	121 ± 24	10	0.033	162 ± 37	131 ± 38	9	0.021
HbA1c (%)	9.9 ± 3.2	6.1 ± 0.6	10	0.003	8.0 ± 2.0	7.1 ± 1.0	9	0.158
IDA-A1c (%)	10.7 ± 3.2	6.4 ± 0.9	10	0.002	10.0 ± 2.7	8.4 ± 1.5	9	0.031
Insulin dose (Units/day)	12.5 ± 7.8	3.9 ± 6.7	10	0.009	39.6 ± 18.8	23.8 ± 9.9	9	0.006
IWR (Units/Kg/day)	0.15 [0.10; 0.26]	0.00 [0.00; 0.15]	10	0.028	0.49 [0.31; 0.67)	0.32 [0.22; 0.41]	9	0.007
Number of insulin-free patients	0/10	7/10	10	0.016	0/9	0/9	9	1.000
C-peptide (pmol/L)	190 [136; 269]	470 [214; 713]	8	0.013	33 [33; 159]	33 [33; 126]	9	0.625

Data are values for patients on triple drug combination therapy within 12 months after starting insulin treatment (early therapy, n = 10 patients) and those on triple drug combination therapy more than 12 months after starting insulin treatment (late therapy, n = 9). Data are means ± SD if the distribution of values was close to normal, or medians with interquartile width in parenthesis if the distribution of values differed from normal. Although they had a normal distribution, we used medians with interquartile width in early therapy c-peptide, late therapy weight, and late therapy IWR to reconcile the data between early and late therapy. P-values are levels of significant paired differences between values before and after the drug combination therapy, for the number of patients (n) indicated.

**Figure 2:**
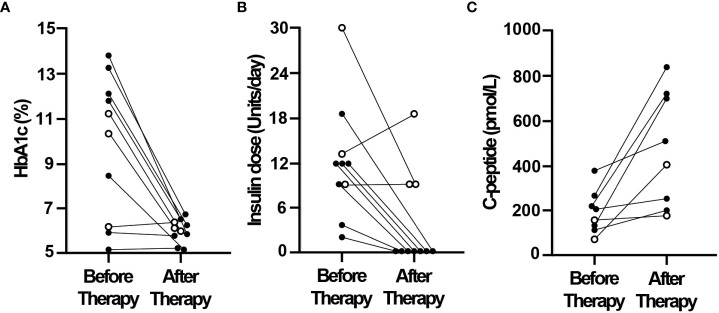
Ten patients were treated early (within 12 months) after diabetes onset and the start of insulin therapy by the addition of a three-drug combination of GABA, a DPP4i and a PPI. HbA1c **(A)**, daily insulin doses **(B)** and plasma C-peptide levels **(C)** are shown for each patient before and after 27-42 weeks of the three-drug combination therapy. Insulin treatment was discontinued in 7 of the 10 patients who had HbA1c levels ≤6.8% after therapy (solid circles) but not in the remaining 3 of the 10 patients (white circles) who continued to require insulin to maintain HbA1c levels <7.0%.

**Figure 3:**
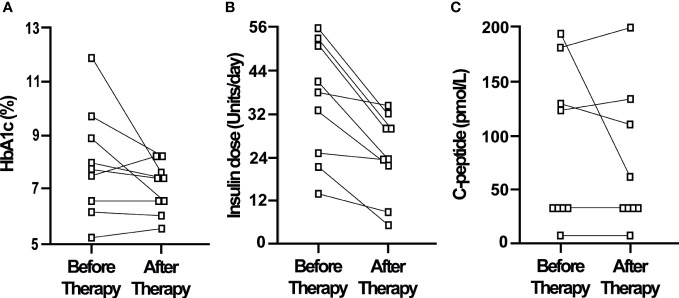
Nine patients were treated late (more than 12 months) after diabetes onset and the start of insulin therapy by the addition of a three-drug combination of GABA, a DPP-4i and a PPI. HbA1c **(A)**, daily insulin doses **(B)** and plasma C-peptide levels **(C)** are shown for each patient before and after therapy with the three-drug combination therapy for a mean of 29 weeks (range 26-41 weeks).

Further examination of the responses of individual patients to CT revealed that insulin treatment could be discontinued in 7 of the 10 patients in the early therapy group, while HbA1c levels were maintained below 7% (mean 6.0%, range 5.2-6.8%) (**Figure 2**). Insulin independence in these 7 patients began at a median of 6 weeks after the start of CT and continued to last until study end at 27-37 weeks. Also, fasting plasma C-peptide levels were increased in all 8 patients in whom it was measured both before and after CT (**Figure 2**). **Figure 3** shows that daily insulin dose requirements were also decreased in all 9 of the late therapy patients, however insulin treatments could not be discontinued. HbA1c levels were decreased in most patients (6 of 9), but HbA1c was <7% in only 4 of these 9 late therapy patients who continued to require insulin treatments to maintain their HbA1c levels <7%. Also, fasting plasma C-peptide levels remained low (<200 pmol/L) in all 9 late therapy patients (**Figure 3**).

**Figure 4:**
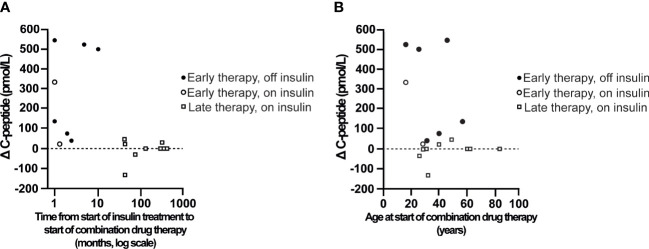
The plasma C-peptide response (delta C-peptide, y-axis) to the combination of GABA, a DPP-4i and a PPI is shown as a function of the time interval from the start of insulin treatment to the start of the combination therapy **(A)**, and as a function of patient age at the start of combination therapy **(B)**. Patients treated with the drug combination within 12 months after the start of insulin treatments were subsequently able to stop insulin treatments (Early therapy, off insulin) or not (Early therapy, on insulin). Other patients treated with the drug combination more than 12 months after the start of insulin treatments were not able to stop insulin treatments (Late therapy, on insulin).

The relationship between the C-peptide response to CT and the time interval from the start of insulin treatment until the start of CT is shown in **Figure 4A**. This shows that C-peptide levels increased in all patients treated with CT within 12 months after the start of insulin treatment, and 6 of these 8 early therapy patients were able to stop taking insulin (paired C-peptide values were available for 8 out of 10 patients). In contrast, C-peptide levels were unchanged in 5 patients, increased in 2 patients, and decreased in 2 patients treated with CT more than 12 months after the start of insulin treatment, and none of these patients were able to discontinue insulin treatment. **Figure 4B** shows that the early therapy patients who were able to discontinue insulin treatments spanned a wide age range (17-58 years).

Addition of the combination of GABA, DPP-4i and PPI to insulin therapy was well tolerated by all patients and no adverse effects were recorded.

## 4 Discussion

In this study, we evaluated the efficacy of concurrent administration of GABA, DPP-4i and PPI as an adjunct combination therapy (CT) to insulin in the management of patients with T1D, in a real-world clinical setting. We found that after 26-42 weeks of this CT, patients with T1D experienced significantly decreased FBG, HbA1c, IDA-A1c and insulin requirements, while fasting plasma C-peptide levels were significantly increased. Importantly, patients who received the CT within 12 months after starting insulin treatment (early therapy) experienced better outcomes than patients who received the CT more than 12 months after starting insulin treatment (late therapy). Daily insulin doses and IWR decreased to lower levels in patients who received early rather than late therapy, and 7 of the 10 patients who received early CT were able to discontinue insulin treatment and maintain good glycemic control, as evidenced by HbA1c levels of 5.2-6.8% in these patients. A HbA1c level of 7% is considered the target for adequate glycemic control in most adults with T1D (17). In addition, the fasting plasma C-peptide level was significantly increased in early therapy patients, whereas it was very low at baseline before therapy and remained low after treatment in late therapy patients. The very low fasting plasma C-peptide level at baseline before CT in the late therapy patients, compared with the early therapy patients, reflects the progressive loss of islet β-cell function that occurs over months without therapy, other than insulin, in patients with T1D (18). Therefore, the significant increase of fasting plasma C-peptide observed in patients who received early treatment with the CT appears to represent an improvement of islet β-cell function.

Interestingly, patients who received CT late after the start of insulin treatment still experienced significant decreases in FBG, IDA-A1c, daily insulin use and IWR without any increase in already very low fasting plasma C-peptide levels. This suggests an additional islet β-cell independent action of the CT to improve glycemic control. Possible actions include decreased glucagon secretion by islet α-cells, a reported action of GABA (15, 19) as well as that of GLP-1 (20), whose blood level is raised by DPP-4 inhibitors. In addition, GABA has been reported to increase insulin sensitivity (21).

The above clinical outcomes reported here are consistent with our findings in a recent study of this combination of GABA, a DPP-4i, and a PPI in NOD mice, an animal model for human T1D (16). Importantly, the combination of all three drugs prevented and reversed autoimmune diabetes in the NOD mice, whereas all two-drug combinations were less or not effective. The improved efficacy of the three components administered together may be explained by synergies of the complex cellular mechanisms of action of the individual components. DPP-4i (22, 23), PPI (12) and GABA (19) have been reported to target different processes considered to be involved in the pathogenesis of islet β-cell destruction and consequent T1D development.

DPP-4 is an enzyme strongly expressed on pancreatic islet cells and on the surface of immune cells (24). DPP-4i, such as sitagliptin or saxagliptin used in this study, prevent degradation of the incretin hormone GLP-1, thereby prolonging the stimulation of GLP-1 receptors. This leads to sustained elevation of cAMP and activation of the PI3K/AKT signaling pathway, which together activate key transcription factors for β-cell function, survival and growth (25). GLP-1 lowers blood glucose levels by stimulating insulin secretion from pancreatic β-cells in a glucose-dependent manner, while simultaneously inhibiting glucagon secretion from α-cells (20). In addition, DPP-4i has anti-inflammatory and immunomodulatory properties that may be beneficial in targeting autoimmune mechanisms operating in T1D. For example, inhibition of membrane and serum DPP-4 by DPP-4i leads to decreased T-cell activation, proliferation, and migration, as well as increased GLP-1-mediated uptake of GABA by immune and endocrine cells (26). A meta-analysis of studies of the combination of a DPP-4i and insulin, in adult patients with latent autoimmune diabetes, reported improved glycemic control, increased islet beta-cell cell function, and decreased incidence of hypoglycemia compared with insulin treatment alone (27). However, a systematic review on the use of DPP-4i in five randomized controlled trials in patients with T1D did not strongly support its beneficial effects, although some improvements were observed in HbA1c levels (14).

PPI drugs, such as omeprazole, the other drug used in this study, increase endogenous gastrin levels, and gastrin was reported to stimulate β-cell regeneration and improve glucose tolerance in 95% pancreatectomized rats (28). Furthermore, a PPI could simultaneously increase insulin secretion by inhibiting the V-ATPase proton pump in β-cell insulin granules (29). Combination of GLP-1 with gastrin (30), as well as the combination of a DPP-4i and a PPI (31), have been reported to increase islet β-cell mass and restore normoglycemia in NOD mice with autoimmune diabetes.

The combination of a DPP-4i and a PPI was studied in a 12-month prospective, placebo-controlled phase 2 trial in patients with recent onset T1D (32). No significant improvements were observed in plasma C-peptide, HbA1c or insulin requirements. However, contrary to expectations, blood levels of GLP-1 and gastrin were found to be elevated in only a portion (45%) of the patients treated with the DPP-4i and PPI combination (32). Therefore, it remains to be determined if the expected elevations of GLP-1 and gastrin can be achieved in response to DPP-4i and PPI drugs, respectively, in most or all patients with T1D, and whether this might then be therapeutic.

In this study, we assessed the therapeutic potential of a Generally Recognized as Safe (GRAS) – approved dietary supplement of GABA, added as a third component to the combination of a DPP-4i and a PPI. GABA functions as an important transmitter within the pancreatic islets and was found to promote islet β-cell regeneration and to protect β-cells against apoptosis induced by cytokines, drugs, and other stressors (19). GABA also has anti-inflammatory and immunoregulatory effects, potentially targeting autoimmune factors present in T1D (19, 33).

The combination of GABA and DPP-4i has been reported to promote regeneration of β-cells and reduce their apoptosis in the mouse model of streptozotocin (STZ)-induced β-cell injury (34) and in human islets transplanted into immunodeficient mice with STZ-induced diabetes (35). These results were reported to be due, in part, to an additive effect of the agents to activate the PI3K/AKT pathway, stimulate cAMP-β-catenin signaling, reduce TxNIP activity, and promote SIRT1 and α-Klotho expression (26, 36).

We recognize the limitations of the real-world retrospective clinical study reported here. The study was not carried out according to a prospective study protocol with defined enrollment windows and lacked a placebo control group of patients. Also, the sample of a T1D patient population studied was small. In addition, the observational nature of this study limits findings to the accuracy and completeness of information documented in the medical records. For example, two patients out of the total of nineteen did not have a recorded pair of C-peptide measurements.

Nevertheless, this retrospective study found that addition of GABA, a DPP-4i and a PPI combination therapy (CT) to insulin therapy significantly improved glycemic control together with reduced insulin requirements in nineteen patients with T1D. Furthermore, diabetes remission (normal or near normal blood glucose levels in the absence of insulin therapy) was observed in 7 out of 10 patients started on the adjunct CT within 12 months after the start of insulin therapy. In comparison, a definitive prospective study of diabetes remission in 268 consecutive patients with new-onset T1D, mostly adults, reported a rate of 18.3% (median 6 months) for partial remission from insulin therapy and a complete remission rate of 12.3% (median 6 months) (37). This indicates that the beneficial effects on glycemic control in recent-onset T1D patients, by the addition of CT to insulin therapy in our study, exceeds what might otherwise occur with insulin therapy alone. In addition, the late therapy T1D patient cohort experienced a significant decrease in daily insulin use and insulin dose-adjusted HbA1c, which could be classified as partial remission and is highly unusual in established T1D patients. A follow-up of the patients in our study is required in order to determine the long-term effects of this combination of GABA, a DPP-4i and a PPI.

GABA, a DPP-4i and a PPI are well-known, safe, readily available, and affordable compounds, thereby providing a practical and promising treatment for T1D. Larger prospective, randomized, placebo-controlled clinical trials are warranted in order to assess the efficacy of adding GABA, a DPP-4i and a PPI to insulin therapy as adjunct treatment for improved glycemic control, together with reduction of insulin requirements in patients with recent onset T1D.

## Data availability statement

The raw data supporting the conclusions of this article will be made available by the authors, without undue reservation.

## Ethics statement

The studies involving human participants were reviewed and approved by Helsinki Ethics Committee of Assuta Medical Centers. The patients/participants provided their written informed consent to participate in this study.

## Author contributions

Conceptualization: DK, LK, and SL. Data curation: FL-R, DK, LK, AR, and SL. Formal analysis: FL-R, DK, LK, AR, and SL. Investigation: FL-R, DK, LK, AR, HS, and SL. Methodology: FL-R, DK, AR and LK. Project administration: DK, LK, SL, and HS. Resources: DK, LK, HS, and SL. Supervision: FL-R, DK, LK, AR, HS, and SL. Validation: FL-R, DK, AR, LK, HS, and SL. Writing—original draft: FL-R, DK, LK, AR, HS, and SL. Writing—review and editing: FL-R, DK, LK, AR, HS, and SL. All authors contributed to the article and approved the submitted version.
